# Perceived control and support during childbirth and their association with postpartum mental health outcomes: a cross-sectional study

**DOI:** 10.3389/fpsyg.2026.1838163

**Published:** 2026-07-22

**Authors:** Rocío Adriana Peinado-Molina, Antonio Hernández-Martínez, Leticia Molina-García, Sergio Martínez-Vázquez

**Affiliations:** 1 Department of Nursing, University of Jaén, Jaén, Spain; 2 Consortium for Biomedical Research in Epidemiology and Public Health (CIBERESP), Madrid, Spain; 3 Department of Nursing, Physiotherapy, and Occupational Therapy, Faculty of Nursing, University of Castilla-La Mancha, Ciudad Real, Spain; 4 Research Group on Care and Evidence-Based Practice, Castilla-La Mancha Health Research Institute (IDISCAM), Ciudad Real, Spain; 5 University Hospital of Jaén, Jaén, Spain

**Keywords:** birth plan, perceived control, postpartum depression, post-traumatic stress disorder, SCIB scale, suicide

## Abstract

**Background:**

The perinatal period is a highly vulnerable stage for maternal mental health, during which anxiety, postpartum depression (PPD), postpartum post-traumatic stress disorder (PTSD) and suicide risk may arise. Perceived maternal control and support during childbirth are important dimensions of the childbirth experience and may be associated with postpartum mental health outcomes.

**Objective:**

To analyze the association between perceived control and support during childbirth and the suicide risk, PPD, anxiety, and PTSD in Spanish women in the postpartum period.

**Method:**

A cross-sectional study was conducted with 302 women who had given birth in the last 6 months. Validated instruments were used: The Support and Control in Birth (SCIB) scale to assess the degree of support and control; the Perinatal Post-traumatic Stress Disorder Questionnaire (PPQ) to measure PTSD; the Edinburgh Postnatal Depression Scale (EPDS) to identify PPD; the Generalized Anxiety Disorder Scale (GAD-7) to assess anxiety; and the Paykel Scale to estimate suicide risk. Bivariate and multivariate analyses were performed using logistic regression, estimating odds ratios (OR) and adjusted odds ratios (aOR) and 95% confidence intervals.

**Results:**

Higher SCIB scores were associated with lower odds of PTSD (aOR = 0.97; 95% CI: 0.96–0.99). Failure to respect the birth plan was associated with higher odds of PTSD (aOR = 3.11; 95% CI: 1.23–7.89). Multiparity was associated with lower odds of postpartum PTSD symptoms (aOR = 0.32; 95% CI: 0.13–0.80), while early initiation of breastfeeding was associated with lower odds of postpartum PTSD symptoms (aOR = 0.37; 95% CI: 0.18–0.78). Conversely, health problems during pregnancy were associated with higher odds of PPD symptoms (aOR = 2.03; 95% CI: 1.00–4.12). No significant associations were found between SCIB and risk of suicide, PPD, or anxiety. Suicide risk was linked to a history of abortion and neonatal ICU admission, PPD to recent stressful events, and anxiety to gestational problems and a history of mental health treatment.

**Conclusion:**

Higher perceived control and support during childbirth were independently associated with lower odds of postpartum post-traumatic stress disorder symptoms. No independent associations were observed between SCIB scores and suicide risk, postpartum depression or anxiety. These findings suggest that perceived support and control during childbirth may be relevant dimensions of the postpartum psychological experience, particularly in relation to postpartum post-traumatic stress symptoms.

## Highlights

- High perceived control and support during childbirth, reflected by higher SCIB scores, were related to lower odds of postpartum post-traumatic stress disorder.
- Lack of respect for the birth plan was the main factor related to postpartum post-traumatic stress disorder.
- Respectful childbirth care, centered on maternal autonomy and emotional support, should be strengthened in perinatal services.

## Introduction

The perinatal period, which spans from pregnancy through the first year postpartum, represents a stage of particular biopsychosocial vulnerability in a woman’s life (World Health Organization, 2022). During this period of physical, hormonal, and social changes, maternal mental health can be significantly affected, and women may experience various disorders that negatively impact both their individual well-being and the development and establishment of early bonding with their newborn (Goodman and Tyer-Viola, 2010; Schalla and Stengel, 2024).

Anxiety, postpartum depression (PPD), postpartum post-traumatic stress disorder (PTSD), and suicide risk are problems that can frequently occur during this stage (World Health Organization, 2020). It is estimated that postpartum anxiety and depression affect 10% of women in developed countries (Dennis et al., 2017; Woody et al., 2017)and the risk of postpartum post-traumatic stress disorder lies between 11.1 and 12.7% (Hernández-Martínez et al., 2021; Martínez-Vázquez et al., 2021a,b). Furthermore, these maternal mental health conditions are related to suicidal ideation, a problem that has been on the rise in recent years (De Backer et al., 2023; Reis, 2025). In Spain, the prevalence of suicidal ideation has been reported to be 12.7–19.3%, and that of suicide attempts 2.4% (Martínez-Galiano et al., 2024; Martínez-Vázquez et al., 2024).

Women who already have a history of mental health problems may experience a worsening of their symptoms or may develop these conditions for the first time during the postpartum period (World Health Organization, 2022). The onset or worsening of mental health problems during this period can have a significant impact on the woman, the baby, and the family environment. This situation not only affects their emotional well-being, but also increases the risk of obstetric complications such as pre-eclampsia, postpartum hemorrhage, and premature birth (Alder et al., 2007; Grote et al., 2010; Howard and Khalifeh, 2020). It can also influence the health of the baby, increasing the likelihood of low birth weight, physical illness, and even difficulties in emotional and behavioral development (Alder et al., 2007; Barker et al., 2012; Dunkel Schetter and Tanner, 2012; Field et al., 2010; Howard and Khalifeh, 2020).

Several factors have been identified that are associated with these mental health disorders, including traumatic experiences during childbirth, gender-based violence, harmful habits, low educational attainment, poor maternal nutrition, lack of social support (Ayers et al., 2016; O’hara and Swain, 1996; Organización Panamericana de la Salud, 2023), as well as the woman’s perception of maternal control, support, and satisfaction with childbirth (Ford et al., 2009; Liu et al., 2020).

Perceived control during childbirth is the woman’s psychological sense of being able to make decisions, manage pain autonomously, and maintain self-efficacy in the clinical setting, which is related to an internal locus of control (Chabbert et al., 2021; Danish et al., 2025; Liu et al., 2020). On the other hand, Perceived Support refers to the influence of interpersonal factors, both medical and psychological, that modulate the birth experience, where the perception of safety and respect prevents stressful clinical events from generating a negative experience (Ford et al., 2009; Liu et al., 2020).

The perception of childbirth is a complex construct, influenced by obstetric, psychosocial, and interpersonal factors (Danish et al., 2025). Within this construct, the feeling of maternal control stands out as the most important variable for satisfaction during childbirth and a key factor for maternal self-esteem (Ford et al., 2009). Its loss can significantly decrease self-esteem and generate negative self-concepts (Díaz Ogallar et al., 2025; Lewkowitz et al., 2024). Maternal satisfaction has been found to depend on multiple modifiable factors, notably decisions made during childbirth, pain management, quality of communication, and support received from healthcare professionals (Demirel et al., 2022; Haines et al., 2013; Liu et al., 2020). Therefore, maternal control during childbirth, perceived support, and satisfaction are fundamental constructs that must be measured reliably, and validated tools are available in Spain (Martínez-Vázquez et al., 2026).

Despite the evidence on the importance of the childbirth experience, there is still a specific evidence gap regarding how the feeling of maternal control and perceived support influence the risks of mental health disorders. Therefore, this study proposes to analyze the relationship between maternal control and perceived support during childbirth and postpartum mental health outcomes, including postpartum depression, anxiety, postpartum post-traumatic stress disorder, and suicide risk.

## Methodology

### Design and subject selection

A cross-sectional study was conducted with women in Spain during the postpartum period who met the following inclusion criteria: being in the postpartum period and having given birth less than 6 months ago. Women under the age of 18 and those who did not understand Spanish (language barrier) were excluded.

The maximum modeling criterion was used to estimate the sample size, which requires a minimum of 10 subjects with the event for each independent variable (Peduzzi et al., 1996). For this calculation, we used the risk of PTSD as the main outcome variable, which reaches prevalences that could be as high as 23.5% (Hernández-Martínez et al., 2021; Liu et al., 2020). To maintain the statistical power and stability of the multivariate model, inclusion was limited to a maximum of 7 independent variables (75 cases/10 events per variable).

### Information source and study variables

The information was collected using the medical records of the women in the study and an *ad hoc* information collection questionnaire, which had been piloted previously. This questionnaire compiled all the variables and instruments used to carry out systematic information collection. It includes instruments such as the Support and Control in Birth (SCIB) scale. This scale consists of 33 items that assess internal and external maternal control, as well as perceived support (Ford et al., 2009), and has been validated for the sociodemographic context of the sample, showing excellent internal consistency (Cronbach’s alpha = 0.951) (Martínez-Vázquez et al., 2026). The modified Perinatal Post-traumatic Stress Disorder Questionnaire (PPQ) on post-traumatic stress was also used. This is an instrument consisting of 14 items that assesses post-traumatic symptoms related to the birth experience, including intrusion or re-experiencing, avoidance behaviors, and hyperactivity or numbing of responsiveness (Hernández-Martínez et al., 2021), with a Cronbach’s alpha of 0.896 in its Spanish validation. The Edinburgh Postnatal Depression Scale (EPDS), consisting of 10 self-administered items, is used to specifically detect postpartum depression (Cox et al., 1987) and has been translated, adapted, and validated in a population of Spanish postpartum women (Garcia-Esteve et al., 2003), showing good internal consistency in recent studies with similar sample (Cronbach’s alpha = 0.87)(Domínguez-Salas et al., 2026). Other tools also included were the Generalized Anxiety Disorder Scale (GAD-7). This scale assesses the presence of anxiety in women (Gómez-Gómez et al., 2024; Löwe et al., 2008) and showed good internal consistency (Cronbach’s alpha = 0.88). Paykel’s suicide risk scale, which consists of five items and assesses suicidal ideation and attempts (Martínez-Galiano et al., 2024; Paykel et al., 1974), with a Cronbach’s alpha of 0.766 in the Spanish validation, both of which were validated in the context of the sample. The variables extracted from the medical records were: sociodemographic variables, obstetric history, variables related to the current delivery, obstetric practices performed, and neonatal outcomes.

The questionnaire was distributed by collaborating healthcare personnel in various care settings: postpartum and delivery wards in healthcare centers, and midwife consultations in health centers. To participate, women were first informed and had to give their informed consent. Once accepted, they were provided with a file containing detailed instructions for completing the questionnaire. To resolve any doubts or queries during the process, participants had two support mechanisms available: a WhatsApp support group and direct assistance from the healthcare professional who invited them to participate.

### Statistical analysis

Absolute and relative frequencies were used to describe the sample for qualitative variables, and the mean and standard deviation (SD) were used for quantitative variables. A bivariate and multivariate analysis was then performed between the scores of the Support and Control in Birth (SCIB) assessment tool, the variables associated with it, and the scores obtained in the PPQ, EPDS, GAD-7, Paykel, and quality of life questionnaires. The scores of the PPQ, EPDS, GAD-7 and Paykel scales were classified according to their validated cut-off points to define dichotomous outcomes indicating the presence or absence of postpartum post-traumatic stress symptoms, postpartum depression risk, anxiety symptoms and suicide risk, respectively. Binary logistic regression models were then fitted for each dichotomous outcome. The SCIB score was included as a continuous explanatory variable. Odds ratios (OR) were estimated in the bivariate analyses and adjusted odds ratios (aOR) in the multivariable analyses, with their respective 95% confidence intervals.

All participants completed the main psychometric questionnaires; therefore, no missing data were observed for the main mental health outcomes. Candidate covariates were selected based on previous evidence, clinical plausibility and their potential role as confounding factors. Final multivariable models were obtained using backward stepwise logistic regression, while retaining clinically relevant variables when appropriate. Model performance and diagnostics were assessed using the Hosmer–Lemeshow goodness-of-fit test and the classification table. Multicollinearity among predictors was examined using variance inflation factors or tolerance values before fitting the final models. The statistical program used for the analysis was SPSS 29.0.

## Results

A total of 302 postpartum women participated, with a mean age of 35.2 years (SD = 4.18); 70.2% (212) were primiparous and 29.8% (90) were multiparous. Forty-nine percent (148) did not profess any religion. 39.4% (119) had had health problems during pregnancy. 35.4% (107) had a birth plan that was respected, and 12.6% (38) had a birth plan that was not respected. 17.9% (54) had a family history of suicide, and 37.4% (113) had experienced a stressful event in the last year. 58.6% (177) had undergone previous treatment related to their mental health. 12.6% (38) did not engage in skin-to-skin contact, and 21.1% (64) did not initiate breastfeeding early. The mean score on the SCIB scale was 113.86 (SD = 27.91). The remaining sociodemographic data for the sample are shown in Table 1.

**Table 1:** Sociodemographic variables.

Variable	Total	Variable	Total
	*N* (%)		*N* (%)
Maternal age		Number of pregnancy visits	
Mean (SD)	35.2 (4.18)	<7	95 (31.5)
Relationship status		7–10	138 (45.7)
Single	53 (17.5)	>10	69 (22.8)
Married	199 (65.9)	Satisfaction degree with pregnancy follow-up	
Domestic partner	46 (15.2)	Not at all	5 (1.7)
Divorced	3 (1.0)	Low satisfaction	31 (10.3)
Widowed	1 (0.3)	Satisfied	85 (28.1)
Nationality		Quite satisfied	77 (25.5)
Spanish	292 (96.7)	Highly satisfied	104 (34.4)
Other	10 (3.3)	Worked during last pregnancy	
Education level		No	50 (16.6)
Primary school	1 (0.3)	Yes	252 (83.4)
Secondary school (not finished)	2 (0.7)	Alcohol consumption during last pregnancy	
Secondary school	7 (2.3)	Never	169 (56.0)
High school	2 (0.7)	Occasionally	113 (37.4)
Professional occupation/Vocational training	33 (10.9)	Frequently	12 (4.0)
University	257 (85.1)	Missing	8 (2.6)
Religious affiliation		Health issues during last pregnancy	
No	148 (49.0)	No	183 (60.6)
Yes, but not practicing	104 (34.4)	Yes	119 (39.4)
Yes, and practicing	50 (16.6)	Hypertension	
Current medical treatment		No	195 (64.6)
No	242 (80.1)	Yes	19 (6.3)
Yes	60 (19.9)	Unsure	0 (0.0)
Family history of suicide		Gestational diabetes mellitus	
No	248 (82.1)	No	191 (63.2)
Yes	54 (17.9)	Yes	26 (8.6)
Stressful event during last year		Unsure	1 (0.3)
No	189 (62.6)	Anemia	
Yes	113 (37.4)	No	143 (47.4)
Current illness		Yes	83 (27.5)
No	241 (79.8)	Unsure	1 (0.3)
Yes	61 (20.2)	Genitourinary infection	
Epidural		No	181 (59.9)
No, maternal choice	31 (10.3)	Yes	41 (13.6)
No, medical reason	4 (1.3)	Unsure	1 (0.3)
No, other reasons	18 (6.0)	Premature birth (risk)	
Yes	249 (82.5)	No	203 (67.2)
Non-pharmacological analgesia		Yes	9 (3.0)
No	197 (65.2)	Unsure	1 (0.3)
Yes	105 (34.8)	Birth plan	
Active participation during birth		No	157 (52.0)
No, by choice	9 (3.0)	Yes, respected	107 (35.4)
No, not possible	67 (22.2)	Yes, but not respected	38 (12.6)
Yes	226 (74.8)	Type of birth	
Perineal tear		Vaginal	181 (59.9)
No	122 (40.4)	Instrumental	55 (18.2)
Episiotomy	49 (16.2)	Elective cesarean section	15 (5.0)
Spontaneous tear (any degree)	130 (43.0)	Emergency cesarean section	51 (16.9)
Episiotomy + tear	1 (0.3)	Satisfaction degree with postpartum care	
Postpartum complications		Not at all	7 (2.3)
No	249 (82.5)	Low satisfaction	28 (9.3)
Yes	53 (17.5)	Satisfied	54 (17.9)
Neonate needed special care		Quite satisfied	80 (26.5)
No	263 (87.1)	Highly satisfied	133 (44.0)
Yes, neonatal intensive care unit	8 (2.6)	Parity	
Yes, neonatology ward	31 (10.3)	Primiparous	212 (70.2)
Early breastfeeding		Multiparous	90 (29.8)
No	64 (21.2)	Any health issue present in last baby born	
Yes	238 (78.8)	No	253 (83.8)
		Yes	49 (16.2)

SCIB; mean (SD) 113.86 (27.91); internal control 34.18 (8.68); external control 35.31 (11.82); support 44.37 (12.27).

### SCIB and suicide risk

No significant relationships were found between SCIB and suicide risk in this analysis, evaluated using the Paykel scale. However, other associated factors were: professing a religion and being a practicing believer (*p* = 0.005; aOR 95% CI: 3.73 (1.49, 9.33)), as well as having had a previous abortion (*p* = 0.032; aOR 95% CI: 9.69 (1.21, 77.60)), the baby being admitted to the neonatal ICU (*p* = 0.020; aOR 95% CI: 8.35 (1.39, 50.02)) or PPD risk (*p* ≤ 0.001; aOR 95% CI: 9.20 (4.07, 20.80)). The remaining information is shown in Table 2.

**Table 2:** Factors associated with perinatal mental health issues: suicide risk, bivariate, and multivariate analysis.

Variable	Suicidal ideation (Paykel Scale)		Bivariate analysis		Multivariate analysis	
	No *n* (%)	Yes *n* (%)	*p*-value	OR 95% CI	*p*-value	aOR 95% CI:
SCIB			**0.007**	**1 (ref.)**		
Mean (SD)	115.61 (27.08)	103.00 (30.79)		0.99 (0.97, 1.00)		
Age			**0.726**	**1 (ref.)**		
Mean (SD)	35.23 (4.27)	34.99 (3.62)		0.99 (0.91, 1.07)		
Religious affiliation						
No	126 (85.1)	22 (14.9)	**0.002**	1 (ref.)	**0.001**	1 (ref.)
Yes, but not practicing	98 (94.2)	6 (5.8)	0.029	0.35 (0.14, 0.90)	0.080	0.40 (0.15, 1.11)
Yes, and practicing	36 (72.0)	14 (28.0)	**0.040**	2.23 (1.04, 4.79)	**0.005**	**3.73 (1.49, 9.33)**
Monthly income (€)						
<1,000	7 (77.8)	2 (22.2)	0.981	1 (ref.)		
1,000–1999	50 (70.8)	14 (21.9)	0.281	0.98 (0.18, 5.26)		
2000–2,999	81 (90.0)	9 (10.0)	0.394	0.39 (0.07, 2.16)		
>3,000	122 (87.8)	17 (12.2)	0.158	0.49 (0.09, 2.54)		
Parity						
Primiparous	181 (85.4)	31 (14.6)	0.582	1 (ref.)		
Multiparous	79 (87.8)	11 (12.2)		0.81 (0.39, 1.70)		
Stillbirth (previous)						
No	256 (86.5)	40 (13.5)	0.188	1 (ref.)	**0.032**	1 (ref.)
Yes	4 (66.7)	2 (33.3)		3.20 (0.57, 18.05)		**9.69 (1.21, 77.60)**
Desired pregnancy						
No	22 (75.9)	7 (24.1)	0.101	1 (ref.)		
Yes	238 (87.2)	35 (12.8)		0.46 (0.18, 1.16)		
Health issues during last pregnancy						
No	162 (88.5)	21 (11.5)	0.132	1 (ref.)		
Yes	98 (82.4)	21 (17.6)		1.65 (0.86, 3.18)		
Birth plan						
No	138 (87.9)	19 (12.1)	**0.022**	1 (ref.)		
Yes, respected	95 (88.8)	12 (11.2)	0.826	0.92 (0.43, 1.98)		
Yes, but not respected	27 (71.1)	11 (28.9)	**0.012**	**2.96 (1.27, 6.92)**		
Type of birth						
Vaginal	161 (89.0)	20 (11.0)	0.068	1 (ref.)		
Instrumental	48 (87.3)	7 (12.7)	0.732	1.17 (0.47, 2.94)		
Cesarean section	51 (77.3)	15 (22.7)	**0.022**	**2.37 (1.13, 4.96)**		
Postpartum complications						
No	219 (88.0)	30 (12.0)	**0.047**	1 (ref.)		
Yes	41 (77.4)	12 (22.6)		**2.14 (1.01, 4.51)**		
Neonate needed special care						
No	20 (87.5)	33 (12.5)	0.113	1 (ref.)	0.067	1 (ref.)
Yes, neonatal intensive care unit	5 (62.5)	3 (37.5)	0.058	4.18 (0.96, 18.32)	**0.020**	**8.35 (1.39, 50.02)**
Yes, neonatology ward	25 (80.6)	6 (19.4)	0.295	1.67 (0.64, 4.38)	0.974	1.02 (0.33, 3.18)
Any health issue present in last baby born						
No	222 (87.7)	31 (12.3)	0.063	1 (ref.)		
Yes	38 (77.6)	11 (22.4)		2.07 (0.96, 4.47)		
Skin-to-skin						
No	30 (78.9)	8 (21.1)	0.178	1 (ref.)		
Yes	230 (87.1)	34 (12.9)		0.55 (0.24, 1.31)		
Early breastfeeding						
No	49 (76.6)	15 (23.4)	**0.015**	1 (ref.)		
Yes	211 (88.7)	27 (11.3)		**0.42 (0.21, 0.85)**		
Family history of suicide						
No	217 (87.5)	31 (12.5)	0.134	1 (ref.)	0.063	1 (ref.)
Yes	43 (79.6)	11 (20.4)		1.79 (0.84, 3.84)		2.36 (0.96, 5.83)
Stressful event during last year						
No	171 (90.5)	18 (9.5)	**0.005**	1 (ref.)		
Yes	89 (78.8)	24 (21.2)		**2.56 (1.32, 4.97)**		
Any previous mental health treatment						
No	113 (90.4)	12 (9.6)	0.073	1 (ref.)		
Yes	147 (83.1)	30 (16.9)		1.92 (0.94, 3.92)		
Admission to a mental health unit						
No	256 (85.9)	42 (14.1)	0.999	1 (ref.)	0.999	1 (ref.)
Yes	4 (100.0)	0 (0.0)		0.00 (0.00, 0.00)		0.00 (0.00, 0.00)
PPD risk						
No	194 (94.2)	12 (5.8)	**<0.001**	1 (ref.)	**<0.001**	1 (ref.)
Yes	66 (68.8)	30 (31.3)		**7.35 (3.56, 15.18)**		**9.20 (4.07, 20.80)**

In bold: statistically significant differences. NC, not calculated; OR, odds ratio; aOR, adjusted odds ratio; CI, confidence interval. Adjusted by SCIB, Family history of suicide, Stressful event during last year, any previous mental health treatment, Stillbirth (previous), Monthly income, Skin-to-skin, Early breastfeeding, Birth plan, PPD Risk, Neonatal current health issue or admission/care in any pediatric unit.

### SCIB and postpartum depression

No significant relationships were found between SCIB and postpartum depression as assessed by the EPDS; however, early breastfeeding was shown to be a protective factor (*p* = 0.015; aOR 95% CI: 0.46 (0.24, 0.86)), and having had a previous stressful event in the last year was identified as a risk factor (*p* = 0.029; aOR 95% CI: 1.82 (1.06, 3.13)). The remaining information is shown in Table 3.

**Table 3:** Factors associated with perinatal mental health issues: postpartum depression, bivariate, and multivariate analysis.

Variable	Postpartum depression (EPDS Scale)		Bivariate analysis		Multivariate analysis	
	No *n* (%)	Yes *n* (%)	*p*-value	OR 95% CI	*p*-value	aOR 95% CI
SCIB			**<0.001**	**1 (ref.)**	**0.014**	**1 (ref.)**
Mean (SD)	118.02 (26.30)	104.93 (29.30)		**0.98 (0.98, 0.99)**		0.99 (0.99, 1.00)
Age			0.094	1 (ref.)	**0.040**	1 (ref.)
Mean (SD)	35.47 (4.34)	34.60 (3.77)		0.95 (0.90, 1.01)		0.93 (0.87, 1.00)
Religious affiliation						
No	97 (65.5)	51 (34.5)	0.422	1 (ref.)		
Yes, but not practicing	76 (73.1)	28 (26.9)	0.205	0.70 (0.40, 1.22)		
Yes, and practicing	33 (66.0)	17 (34.0)	0.953	0.98 (0.50, 1.93)		
Monthly income (€)						
<1,000	4 (44.4)	5 (55.6)	0.194	1 (ref.)		
1,000–1999	39 (60.9)	25 (39.1)	0.352	0.51 (0.13, 2.10)		
2,000–2,999	65 (72.2)	25 (27.8)	0.097	0.31 (0.08, 1.24)		
>3,000	98 (70.5)	41 (29.5)	0.116	0.34 (0.09, 1.31)		
Parity						
Primiparous	137 (64.6)	75 (35.4)	**0.041**	1 (ref.)		
Multiparous	69 (76.7)	21 (23.3)		**0.56 (0.32, 0.98)**		
Stillbirth (previous)						
No	202 (68.2)	94 (31.8)	0.935	1 (ref.)		
Yes	4 (66.7)	2 (33.3)		1.07 (0.19, 5.97)		
Desired pregnancy						
No	16 (55.2)	13 (44.8)	0.117	1 (ref.)		
Yes	190 (69.6)	83 (30.4)		0.54 (0.25, 1.17)		
Health issues during last pregnancy						
No	134 (73.2)	49 (26.8)	0.021	1 (ref.)		
Yes	72 (60.5)	47 (39.5)		**1.79 (1.09, 2.92)**		
Birth plan						
No	112 (71.3)	45 (28.7)	0.016	1 (ref.)		
Yes, respected	76 (71.0)	31 (29.0)	0.957	1.02 (0.59, 1.75)		
Yes, but not respected	18 (47.4)	20 (52.6)	**0.006**	**2.77 (1.34, 5.71)**		
Type of birth						
Vaginal	129 (71.3)	52 (28.7)	0.280	1 (ref.)		
Instrumental	37 (67.3)	18 (32.7)	0.570	1.21 (0.63, 2.31)		
Cesarean section	40 (60.6)	26 (39.4)	0.112	1.61 (0.89, 2.91)		
Postpartum complications						
No	180 (72.3)	69 (27.7)	**0.001**	1 (ref.)	0.051	1 (ref.)
Yes	26 (49.1)	27 (50.9)		**2.71 (1.49, 4.97)**		1.91 (1.00, 3.67)
Neonate needed special care						
No	184 (70.0)	79 (30.0)	0.115	1 (ref.)		
Yes, neonatal intensive care unit	6 (75.0)	2 (25.0)	0.760	0.78 (0.15, 3.93)		
Yes, neonatology ward	16 (51.6)	15 (48.4)	0.042	**2.18 (1.03, 4.63)**		
Any health issue present in last baby born						
No	174 (68.8)	79 (31.2)	0.633	1 (ref.)		
Yes	32 (65.3)	17 (34.7)		1.17 (0.61, 2.23)		
Skin-to-skin						
No	19 (50.0)	19 (50.0)	**0.012**	1 (ref.)		
Yes	187 (70.8)	77 (29.2)		**0.41 (0.21, 0.82)**		
Early breastfeeding						
No	33 (51.6)	31 (48.4)	**0.002**	1 (ref.)	**0.015**	1 (ref.)
Yes	173 (72.7)	65 (27.3)		**0.40 (0.23, 0.71)**		**0.46 (0.24, 0.86)**
Family history of suicide						
No	171 (69.0)	77 (31.0)	0.550	1 (ref.)		
Yes	35 (64.8)	19 (35.2)		1.21 (0.65, 2.24)		
Stressful event during last year						
No	143 (75.7)	46 (24.3)	**<0.001**	1 (ref.)	**0.029**	1 (ref.)
Yes	63 (55.8)	50 (44.2)		**2.47 (1.50, 4.06)**		**1.82 (1.06, 3.13)**
Any previous mental health treatment						
No	96 (76.8)	29 (23.2)	**0.008**	1 (ref.)	0.083	1 (ref.)
Yes	110 (62.1)	67 (37.9)		**2.02 (1.21, 3.37)**		1.64 (0.94, 2.87)
Admission to a mental health unit						
No	203 (68.1)	95 (31.9)	0.770	1 (ref.)		
Yes	3 (75.0)	1 (25.0)		0.71 (0.07, 6.94)		

In bold: statistically significant differences. NC, not calculated; OR, odds ratio; aOR, adjusted odds ratio; CI, confidence interval. Adjusted by SCIB, Family history of suicide, Stressful event during last year, any previous mental health treatment, stillbirth (previous), Monthly income, skin-to-skin, early breastfeeding, birth plan, PPD risk, neonatal current health issue or admission/care in any pediatric unit.

### SCIB and anxiety

No significant relationships were found between SCIB and anxiety. On the contrary, other variables appear to be related, specifically older age was shown to be associated as a protective factor (*p* = 0.004; aOR 95% CI: 0.88 (0.81, 0.96)) and as risk factors having had problems during pregnancy (*p* = 0.016; aOR 95% CI: 2.16 (1.16, 4.05)), and having had some type of previous mental health treatment (*p* ≤ 0.001; aOR 95% CI: 4.67 (2.17, 10.04)). The remaining information can be found in Table 4.

**Table 4:** Factors associated with perinatal mental health issues: anxiety, bivariate, and multivariate analysis.

Variable	Anxiety (GAD-7 Scale)		Bivariate analysis		Multivariate analysis	
	No *n* (%)	Yes *n* (%)	*p*-value	OR 95% CI	*p*-value	aOR 95% CI
SCIB			**<0.001**	**1 (ref.)**	**0.006**	**1 (ref.)**
Mean (SD)	117.09 (26.96)	102.78 (28.49)		**0.98 (0.97, 0.99)**		0.98 (0.97, 1.00)
Age			0.011	1 (ref.)	**0.004**	1 (ref.)
Mean (SD)	35.52 (4.17)	34.06 (4.04)		0.92 (0.85, 0.98)		**0.88 (0.81, 0.96)**
Religious affiliation						
No	109 (73.6)	39 (26.4)	0.187	1 (ref.)	0.106	1 (ref.)
Yes, but not practicing	82 (78.8)	22 (21.2)	0.344	0.75 (0.41, 1.36)	0.747	0.89 (0.44, 1.80)
Yes, and practicing	43 (86.0)	7 (14.0)	0.079	0.46 (0.19, 1.10)	**0.036**	**0.34 (0.12, 0.93)**
Monthly income (€)						
<1,000	5 (55.6)	4 (44.4)	0.004	1 (ref.)	0.034	1 (ref.)
1,000–1999	40 (62.5)	24 (37.5)	0.689	0.75 (0.18, 3.07)	0.740	0.74 (0.13, 4.32)
2,000–2,999	73 (81.1)	17 (18.9)	0.088	0.29 (0.07, 1.20)	0.152	0.28 (0.05, 1.60)
>3,000	116 (83.5)	23 (16.5)	0.049	0.25 (0.06, 0.99)	0.151	0.28 (0.05, 1.59)
Parity						
Primiparous	157 (74.1)	55 (25.9)	0.031	1 (ref.)		
Multiparous	77 (85.6)	13 (14.4)		0.48 (0.25, 0.94)		
Stillbirth (previous)						
No	229 (77.4)	67 (22.6)	0.730	1 (ref.)		
Yes	5 (83.3)	1 (16.7)		0.68 (0.08, 5.95)		
Desired pregnancy						
No	21 (72.4)	8 (27.6)	0.493	1 (ref.)		
Yes	213 (78.0)	60 (22.0)		0.74 (0.31, 1.75)		
Health issues during last pregnancy						
No	154 (84.2)	29 (15.8)	**0.001**	1 (ref.)	**0.016**	1 (ref.)
Yes	80 (67.2)	39 (32.8)		**2.59 (1.42, 4.49)**		**2.16 (1.16, 4.05)**
Birth plan						
No neonatal intensive care unit	126 (80.3)	31 (19.7)	0.033	1 (ref.)		
Yes, respected	85 (79.4)	22 (20.6)	0.871	1.05 (0.57, 1.94)		
Yes, but not respected	23 (60.5)	15 (39.5)	0.012	**2.65 (1.24, 5.67)**		
Type of birth						
Vaginal	146 (80.7)	35 (19.3)	0.249	1 (ref.)		
Instrumental	39 (70.9)	16 (29.1)	0.126	1.71 (0.86, 3.41)		
Cesarean section	49 (74.2)	17 (25.8)	0.275	1.45 (0.75, 2.81)		
Postpartum complications						
No	195 (78.3)	54 (21.7)	0.455	1 (ref.)		
Yes	39 (73.6)	14 (26.4)		1.30 (0.66, 2.56)		
Neonate needed special care						
No	206 (78.3)	57 (21.7)	0.334	1 (ref.)		
Yes, neonatal intensive care unit	7 (87.5)	1 (12.5)	0.540	0.52 (0.06, 4.28)		
Yes, neonatology ward	21 (67.7)	10 (32.3)	0.188	1.72 (0.77, 3.86)		
Any health issue present in last baby born						
No	200 (79.1)	53 (20.9)	0.141	1 (ref.)		
Yes	34 (69.4)	15 (30.6)		1.67 (0.84, 3.28)		
Skin-to-skin						
No	25 (65.8)	13 (34.2)	0.069	1 (ref.)		
Yes	209 (79.2)	55 (20.8)		0.51 (0.24, 1.05)		
Early breastfeeding						
No	42 (65.6)	22 (34.4)	0.012	1 (ref.)	0.084	1 (ref.)
Yes	192 (80.7)	46 (19.3)		**0.46 (0.25, 0.84)**		0.51 (0.24, 1.10)
Family history of suicide						
No	195 (78.6)	53 (21.4)	0.309	1 (ref.)		
Yes	39 (72.2)	15 (27.8)		1.42 (0.73, 2.76)		
Stressful event during last year						
No	155 (82.0)	34 (18.0)	0.016	1 (ref.)		
Yes	79 (69.9)	34 (30.1)		**1.96 (1.14, 3.39)**		
Any previous mental health treatment						
No	114 (91.2)	11 (8.8)	**<0.001**	1 (ref.)	**<0.001**	1 (ref.)
Yes	120 (67.8)	57 (32.2)		**4.92 (2.46, 9.86)**		**4.67 (2.17, 10.04)**
Admission to a mental health unit						
No	232 (77.9)	66 (22.1)	0.213	1 (ref.)		
Yes	2 (50.0)	2 (50.0)		3.52 (0.49, 25.43)		

In bold: statistically significant differences. NC, not calculated; OR, odds ratio; aOR, adjusted odds ratio; CI, confidence interval. Adjusted by SCIB, Family history of suicide, Stressful event during last year, any previous mental health treatment, stillbirth (previous), monthly income, skin-to-skin, early breastfeeding, birth plan, PPD risk, neonatal current health issue or admission/care in any pediatric unit.

### SCIB and post-traumatic stress disorder

With regard to factors associated with the risk of PTSD, higher scores for support and control (SCIB scale) were observed as protective factors (*p* ≤ 0.001; aOR 95% CI: 0.97 (0.96, 0.99)), as were multiparity (*p* = 0.015; aOR 95% CI: 0.32 (0.13, 0.80)) and early breastfeeding (*p* = 0.009; aOR 95% CI: 0.37 (0.18, 0.78)). Conversely, when women perceived that their birth plan was not respected, an increase in risk was observed (*p* = 0.017; aOR 95% CI: 3.11 (1.23, 7.89)). The remaining information can be found in Table 5.

**Table 5:** Factors associated with perinatal mental health issues: postpartum post-traumatic stress disorder, bivariate, and multivariate analysis.

Variable	Postpartum post-traumatic stress disorder (PPQ Scale)		Bivariate analysis		Multivariate analysis	
	No *n* (%)	Yes *n* (%)	*p*-value	OR 95% CI	*p*-value	aOR 95% CI
SCIB			**<0.001**	**1 (ref.)**	**<0.001**	**1 (ref.)**
Mean (SD)	119.14 (24.47)	89.08 (29.89)		**0.96 (0.95, 0.97)**		**0.97 (0.96, 0.99)**
Age	34.18 (8.68)	169 (18.9)	0.650	1 (ref.)		
Mean (SD)	35.25 (4.20)	34.96 (3.77)		0.98 (0.92, 1.06)		
Religious affiliation						
No	122 (82.4)	26 (17.6)	0.942	1 (ref.)		
Yes, but not practicing	85 (81.7)	19 (18.3)	0.886	1.05 (0.55, 2.02)		
Yes, and practicing	42 (84.0)	8 (16.0)	0.799	0.89 (0.38, 2.13)		
Monthly income (€)						
<1,000	7 (77.8)	2 (22.2)	0.088	1 (ref.)		
1,000–1999	46 (71.9)	18 (28.1)	0.711	1.37 (0.26, 7.23)		
2,000–2,999	76 (84.4)	14 (15.6)	0.607	0.65 (0.12, 3.43)		
>3,000	120 (86.3)	19 (13.7)	0.482	0.55 (0.11, 2,87)		
Parity						
Primiparous	167 (78.8)	45 (21.2)	0.012	1 (ref.)	**0.015**	1 (ref.)
Multiparous	82 (91.1)	8 (8.9)		**0.36 (0.16, 0.80)**		**0.32 (0.13, 0.80)**
Stillbirth (previous)						
No	244 (82.4)	52 (17.6)	0.954	1 (ref.)		
Yes	5 (83.3)	1 (16.7)		0.94 (0.11, 8.20)		
Desired pregnancy						
No	19 (65.5)	10 (34.5)	0.015	1 (ref.)		
Yes	230 (84.2)	43 (15.8)		**0.36 (0.16, 0.82)**		
Health issues during last pregnancy						
No	159 (86.9)	24 (13.1)	0.013	1 (ref.)	0.049	1 (ref.)
Yes	90 (75.6)	29 (24.4)		**2.14 (1.17, 3.89)**		2.03 (1.00, 4.12)
Birth plan						
No	136 (86.6)	21 (13.4)	**<0.001**	1 (ref.)	0.043	1 (ref.)
Yes, respected	95 (88.8)	12 (11.2)	0.603	0.82 (0.38, 1.74)	0.901	0.95 (0.41, 2.21)
Yes, but not respected	18 (47.4)	20 (52.6)	**<0.001**	**7.20 (3.28, 15.78)**	**0.017**	**3.11 (1.23, 7.89)**
Type of birth						
Vaginal	165 (91.2)	16 (8.8)	**<0.001**	1 (ref.)		
Instrumental	42 (76.4)	13 (23.6)	0.005	**3.19 (1.43, 7.15)**		
Cesarean section	42 (66.3)	24 (36.4)	**<0.001**	**5.89 (2.88, 12.08)**		
Postpartum complications						
No	212 (85.1)	37 (14.9)	0.009	1 (ref.)		
Yes	37 (69.8)	16 (30.2)		**2.48 (1.25, 4.90)**		
Neonate needed special care						
No	222 (84.4)	41 (15.6)	0.069	1 (ref.)		
Yes, neonatal intensive care unit	6 (75.0)	2 (25.0)	0.479	1.81 (0.35, 9.25)		
Yes, neonatology ward	21 (67.7)	10 (32.3)	0.024	**2.58 (1.13, 5.87)**		
Any health issue present in last baby born						
No	208 (82.2)	45 (17.8)	0.806	1 (ref.)		
Yes	41 (83.7)	8 (16.3)		0.90 (0.40, 2.05)		
Skin-to-skin						
No	23 (60.5)	15 (39.5)	**<0.001**	1 (ref.)		
Yes	226 (85.6)	38 (14.4)		**0.26 (0.12, 0.54)**		
Early breastfeeding						
No	41 (64.1)	23 (35.9)	**<0.001**	1 (ref.)	**0.009**	1 (ref.)
Yes	208 (87.4)	30 (12.6)		**0.26 (0.14, 0.49)**		**0.37 (0.18, 0.78)**
Family history of suicide						
No	208 (83.9)	40 (16.1)	0.167	1 (ref.)		
Yes	41 (75.9)	13 (24.1)		1.65 (0.81, 3.35)		
Stressful event during last year						
No	160 (84.7)	29 (15.3)	0.194	1 (ref.)		
Yes	89 (78.8)	24 (21.2)		1.49 (0.82, 2.71)		
Any previous mental health treatment						
No	110 (88.0)	15 (12.0)	0.035	1 (ref.)		
Yes	139 (78.5)	38 (21.5)		**2.01 (1.05, 3.83)**		
Admission to a mental health unit						
No	246 (82.6)	52 (17.4)	0.696	1 (ref.)		
Yes	3 (75.0)	1 (25.0)		1.58 (0.16, 15.46)		

In bold: statistically significant differences. NC, not calculated; OR, odds ratio; aOR, adjusted odds ratio; CI, confidence interval. Adjusted by SCIB, Family history of suicide, Stressful event during last year, any previous mental health treatment, stillbirth (previous), monthly income, skin-to-skin, early breastfeeding, birth plan, PPD risk, neonatal current health issue or admission/care in any pediatric unit.

Model diagnostics did not indicate relevant multicollinearity among predictors, as all VIF values were below the commonly accepted threshold. The Hosmer–Lemeshow goodness-of-fit tests suggested an acceptable fit for the final logistic regression models.

## Discussion

The perception of maternal control and support during childbirth, assessed using the SCIB scale, was mainly related to postpartum post-traumatic stress disorder. No significant associations were observed with postpartum depression, anxiety, or suicide risk. Additionally, other factors were associated with the different maternal mental health outcomes were identified: suicide risk was associated with religious practice, history of abortion, admission of the baby to the neonatal ICU, and risk of PPD. On the other hand, the risk of PPD increased after experiencing previous stressful events, while early breastfeeding acted as a protective factor. Anxiety was more common in women with problems during pregnancy or with previous mental health treatment, with age being a protective factor. Finally, the risk of PTSD increased when the birth plan was not followed, while multiparity and early initiation of breastfeeding were associated with a lower risk.

Selection bias cannot be ruled out, as voluntary recruitment through maternity wards and primary care midwife consultations may have underrepresented women with more severe psychological symptoms or limited access to healthcare services. Although the study sample was broadly comparable to the reference population in terms of age and income level, this does not exclude other sources of selection bias. Due to the observational nature of the study, possible confounding bias was considered. However, this was controlled for in the design by selecting study subjects with appropriate inclusion and exclusion criteria and subsequently adjusting for all variables that could confound the results using multivariate data analysis. We consider that, given the close follow-up of the women and the use of medical records for clinical data, the influence of anamnestic bias may have had little or no influence. The main strength of the study is its novelty and originality, using a recently validated tool in the Spanish to assess support and control during childbirth (Martínez-Vázquez et al., 2026), together with validated instruments for the main postpartum mental health outcomes. Although previous studies have examined postpartum mental health conditions and variables related to support few have analyzed perceived control and support during childbirth together with several postpartum mental health outcomes using validated instruments in the Spanish postpartum context(Garcia-Esteve et al., 2003; Gómez-Gómez et al., 2024; Hernández-Martínez et al., 2021; Martínez-Galiano et al., 2024) Given the number of outcomes and predictors analyzed, the possibility of type I error due to multiple comparisons cannot be excluded. Therefore, secondary associations should be interpreted cautiously and considered exploratory. Some adjusted estimates, particularly those involving small subgroups such as previous pregnancy loss or neonatal intensive care unit admission, showed wide confidence intervals. These results should therefore be interpreted cautiously, as they may reflect limited precision due to the small number of events. Another limitation concerns the stability of the multivariable logistic regression models. Although the sample size was estimated using an events-per-variable criterion, some predictors included in the models were multi-categorical variables and therefore generated more than one estimated parameter. This may have reduced the effective events-per-parameter ratio, particularly for outcomes with fewer events, such as suicide risk and postpartum post-traumatic stress symptoms. Therefore, some adjusted estimates should be interpreted cautiously, especially those with wide confidence intervals or based on small subgroups.

Suicide risk was associated with a history of miscarriage and the admission of the newborn to the neonatal ICU, findings consistent with other studies (Bright et al., 2022; Martínez-Vázquez et al., 2024; Orri et al., 2019). PPD was also shown to be a risk factor for the onset of suicidal ideation, as reported by various authors (Agrawal et al., 2022; Martínez-Vázquez et al., 2024). Women with a history of mental health disorders were more vulnerable to suicidal ideation. Likewise, the risk of PPD is increased by having suffered a previous stressful event, considered by various authors to be one of the strongest predictors of its onset (Evagorou et al., 2016; Gulamani et al., 2013).

The presence of disease, the loss of close relatives, job loss, and experiences of violence are some of the events that, according to van der Zee-van den Berg et al. (2021), represent important milestones in women’s lives. These authors also find that early breastfeeding does not protect against the onset of PPD, a finding that contrasts with our results. In contrast, breastfeeding, especially if started early, has been widely associated as a protective factor against PPD (Dias and Figueiredo, 2015; Pope and Mazmanian, 2016). The World Health Organization (2025) has stated that breastfeeding can have a positive impact on the mental health of the mother and newborn, reducing the risk of postpartum depression and increasing maternal confidence in parenting, as well as strengthening the mother-baby bond.

Women who experienced complications during pregnancy showed a higher risk of developing anxiety, findings that are in line with those of other authors (Ali et al., 2012; Pawluski et al., 2017). The problems women experience throughout pregnancy (whether physical, psychological, or social) can lead to excessive worry or even negative anticipation of childbirth, i.e., the apprehensive expectation that defines anxiety (American Psychiatric Association, 2022).

Along similar lines, we found that women with a previous history of mental health treatment were at greater risk of postpartum anxiety; a finding that is in agreement with other research (Leach et al., 2017). In a study on perinatal anxiety conducted in 2024 in the United Kingdom, it was found that almost 40% of women had a previous history of mental health problems, which underlines the importance of early intervention (Ayers et al., 2024). In our findings, maternal age acts as a protective factor, although this is contrary to the findings of other authors, where anxiety increases as the mother gets older (Ayu et al., 2019; García-Blanco et al., 2017). This finding suggests that women’s life trajectories lead to better adaptation and a greater ability to cope with and modulate responses to anxiety-provoking stimuli. In this regard, cross-sectional evidence among perinatal cohorts identifies avoidant coping strategies as key modifiable risk factors that exacerbate both postpartum anxiety and depression (Motofelea et al., 2026a,b). Furthermore, according to the psychological vulnerability model, lower maternal self-esteem strongly correlates with higher depressive and anxiety symptom scores during the postpartum transition, (Motofelea et al., 2026a,b) underscores the need for validated screening tools like the EPDS and GAD-7 to accurately monitor these multi-faceted mental health trajectories across differing demographics (Motofelea et al., 2025).

The risk of PPTSD was increased in women whose birth plan was not respected, consistent with previous findings (Hernandez-Martínez et al., 2019, 2020; Martínez-Vázquez et al., 2021a,b). Failure to meet these expectations can alter the birth experience, contributing to the development of PTSD symptoms. This clinical deterioration is often triggered when a disregard for maternal autonomy shatters a woman’s sense of agency, forcing a reliance on maladaptive coping mechanisms. A greater perception of self-efficacy during childbirth has been linked to fewer postpartum post-traumatic stress symptoms, whereas negative childbirth experiences have been associated with a higher likelihood of these symptoms (Tomsis et al., 2021). The mother’s perceived self-efficacy during childbirth helps reduce the risk of these symptoms, while negative childbirth experiences have been associated with a predisposition to developing the condition (Inci et al., 2015).

Multiparity was associated with lower odds of PTSD. Swift et al. (2024) found similar results in their study, which included 924 postpartum women. However, other authors found that being a primiparous woman acts as a protective factor, possibly due to the support provided by the midwife, which compensates for the negative effects of being a first-time mother (Sigurdardottir et al., 2017).

Early breastfeeding was associated with a lower odds of developing PTSD symptoms, something that has been described previously in other studies. In addition to acting as a protective factor, the lack of promotion or establishment of breastfeeding may constitute a risk factor (Cook et al., 2018; Martínez-Vázquez et al., 2021a,b).

It is essential that clinical guidelines incorporate the early detection of these factors, such as non-compliance with the birth plan or psychiatric history, from the prenatal check-up stage. Early care focused on promoting a birth environment that prioritizes women’s autonomy and emotional support should be considered an important component of perinatal care. Implementing standardized instruments like the SCIB can help clinicians quantify this perceived control, ensuring that respectful, mother-centered care actively mitigates the development of severe postpartum psychological morbidity.

## Conclusion

Postpartum post-traumatic stress disorder was the maternal mental health outcome linked to perceived control and support during childbirth, as measured with the SCIB scale. Respectful childbirth care, centered on maternal autonomy and emotional support, should remain a priority in perinatal services.

## Data Availability

The original contributions presented in the study are included in the article/supplementary material, further inquiries can be directed to the corresponding author.
